# American Dental Society of Europe

**Published:** 1884-12

**Authors:** W. St. Geo. Elliott

**Affiliations:** London


### AMERICAN DENTAL SOCIETY OF EUROPE.

MEETING AT VEVEY, SWITZERLAND, AUG. 26, 27, AND 28, 1884.

REPORTED FOR THE INDEPENDENT PRACTITIONER BY DR. W. ST. GEO. ELLIOTT, OF LONDON.

### AFTERNOON SESSION.

During the afternoon there was a large attendance at the clinics. Dr. De Trey removed a cement filling from a molar in which the nerve had been resected by his process some eight months previously.

Apparently the pulp was in a healthy condition. In another case the operation of resection was performed, although the patient gave evidence that it was not a painless process. A case was exhibited (a patient of Dr. Wetzel), showing extensive restoration in gold of many of the teeth; also a case of bridge work. Gold fillings were put into the cavities on either side; into the anterior one, one end of the bar containing the interposed tooth was set; a platinum hollow screw or cylinder was built into the filling in the molar; a screw passed vertically through the other end of the bar (here flattened) and was screwed into the cylinder in the molar, thus making a strong, cleanly, and easily removed bridge.

*Dr. De Trey*—Exhibited a case of much interest. The gentleman had broken off a right upper central, a tooth formerly partially girdled with gold. The pulp was dead, and the root gave trouble for a long time. Finally it was discovered that the tip of the root had also been broken by the blow, it making its appearance at the free margin of the gum. The main fracture was nearly transverse at the neck, and was treated by a pin in the natural crown, fastened with cement, crown, pin and all being put in place upon the root. As the labial filling had become detached by the manipulation, an impression was taken in oxy-phosphate; into the impression a gold filling was placed in the exact position of the cavity of the original filling. The impression was then broken, and the gold filling put into its place in the mouth. When examined by the members it appeared exactly as the original must have done, and served to strengthen the tooth.

*Dr. Edwards*—Exhibited a new matrix with applier. It was of steel, shaped like a sickle, forming a double wedge, so that when placed between the teeth near the gum it was pushed up or down as the case required, thus forming a sort of universal matrix.

*Dr. Elliott*—Exhibited a new mallet. The handle, cylinder, etc., were made of a new alloy—bismuth bronze—of silver color. The part held in the hand was mottled celluloid, while between this part and the hammer head the metal was flattened to make a spring. The cylinder was filled with lead, but at one end a thick cushion of leather had been inserted, thus giving great weight to overcome the loss by the softness of the leather. The mallet was pleasanter to the patient than the older forms. He also exhibited some new double-end aluminum bronze plastic instruments, the points not being oxidizable. Also a new syringe made of the hypodermic pistonless syringe, adapted to a handle. These syringes are cheap, costing but two francs, and it is desirable to keep several on hand; one for injecting fluid gutta percha into nerve canals; another for applying the medicaments required in the treatment of pyorrhœa alveolaris; another for alveolar abscesses, etc.

WEDNESDAY, AUGUST 27.

The meeting was called to order, Dr. Edwards, Vice-President, in the chair.

*Dr. Miller*—Briefly discussed some disputed points in dental caries. The following is an abstract: There are many writers on the etiology of dental caries, especially in America, who, not taking the trouble to really investigate or experiment upon the subject, still think they have rendered great service to the profession when they have covered pages with quotations from Cohn, Pasteur and Tyndal, regardless of date, relative to the wonderful effects which may be produced by micro-organisms. It would be advisable for those who wish an insight into the subject of mycology to turn their attention to the much more exact researches of the present German school. Even then, such work as this could never bring us any nearer to an understanding of the phenomena of dental caries. The question is not what bacteria in general have been known to do, or how many of them may dance upon the point of a needle, etc., etc. It is, what *do* the micro-organisms which are found upon or in the teeth do? And all the experiments that have ever been made, unless upon the teeth themselves, could not furnish an answer to the question. We must know the fungi themselves, and their characteristics, and then we may reason intelligently as to what their effect will be when placed under such conditions as are found in the human mouth. In short, we must study *caries dentium* by the same methods by which tuberculosis, pneumonia, cholera, etc., have been studied; i. e., by the method of pure culture. I do not mean, however, to enter into a discussion of these questions here; I wish only to refer briefly to two facts that I have long since established, but which have been lately called in question. These are the possibility of producing caries artificially, and the existence of carious dental tissue which contains no micro-organisms. With regard to the first of these, I can only reiterate what I

have so often said before, that I have produced caries artificially, which is not to be distinguished from natural caries, as Dr. Bödecker and many others can testify.

With regard to the second question, I may say that I have never yet examined a decaying tooth that did not demonstrate the fact referred to. It is often particularly pronounced in cases of extensive decay on the grinding surface. If we break away the overhanging walls of enamel, and scoop out thoroughly the carious dentine in one piece, sections of the same properly stained will show large territories at both ends which do not contain a trace of organisms, simply because they cannot travel perpendicularly to the dentinal tubules as fast as the acids can. At the bottom of the cavity may also be found tracts containing no organisms, though generally not so extensive. Dr. Miller then took a piece of carious dentine from a tooth of the above description, prepared specimens from it, and demonstrated on them as well as on many other preparations, to the satisfaction of all present, the truth of the views set forth. He further exhibited a number of preparations of artificial caries which no one ventured to distinguish from natural caries.

*Dr. Du Bouchet*—What do you consider the best antiseptic?

*Dr. Miller*—I would refer to my papers in the INDEPENDENT PRACTITIONER, one of which gives a list of substances tested for their powers in this respect. However, hydraygrum bi-chloridum is, doubtless, the most potent; but as it is a powerful poison it must be used diluted, say to one per cent.; but it would seem that iodoform fills the requirements as well as it is possible to expect.

*Dr. George*—Does the decalcified portion you have mentioned, in which there are no micro-organisms, become recalcified?

*Dr. Miller*—No doubt it *may*.

*Dr. Terry*—Would it not be safe practice to excavate the decalcified part and fill with cement?

*Dr. George*—But the recalcification is certainly better than any artificial substitute.

*Dr. Miller*—Of course, if in excavating you cut off the tubules from the pulp, you can have no recalcification.

*Dr. Bodecker*—Recalcification can only occur near the pulp. There must of necessity exist a grade of low inflammation, the same as in all cartilaginous parts, and before recalcification can occur there must be a so-called retrograde metamorphosis; that is, there must be a breaking down of the more complex into the more simple elementary combinations, and from this medullary condition advance.

It has afforded me a great deal of pleasure to make as thorough a study as time would permit of a number of the fine preparations of Dr. Miller, both natural and artificial, and I must say that they fully corroborate everything that he has said. I have not, however, as yet been able to give much attention to this subject.

*Dr. Terry*—Has Dr. Miller always found the same fungus?

*Dr. Miller*—No. There are at least five different kinds of fungi which prey upon the human teeth; not, however, all upon one and the same tooth. They can exist without free access of air, and do not appear to be instrumental in producing putrefaction.

*Dr. Terry*—All the varied forms of decay have a cause in organisms, have they not?

*Dr. Miller*—Yes, I think so.

*Dr. Terry*—Is there, then, one which might be considered normal?

*Dr. Miller*—That is a very difficult question; the fungi have a separate existence, and are not the result, but the cause of decay. The latter, so far as the organisms are concerned, is the normal result of their action, but as far as the teeth are concerned we would probably call it an abnormal condition.

*Dr. Terry*—All air, then, contains the organisms. Does the air found at different attitudes or in different places contain different amounts of the fungi? Is the air of the Engadine freer than that of Vevey?

*Dr. Miller*—Yes; undoubtedly temperature, humidity, elevation, etc., are important in determining the numbers, but perhaps humidity is the most important. When moist the organisms stick to anything they may come in contact with, but when they become dry they are released and float in the air.

*Dr. Terry*—The French, in opposition to Dr. Koch's views, water the streets during the cholera epidemic. Are there organisms in the blood?

*Dr. Miller*—In a state of perfect health there probably are not; but when any pathological condition is present there may be. This is my present impression.

(TO BE CONTINUED.)

